# Formation of the Ventricles

**DOI:** 10.1100/tsw.2006.316

**Published:** 2006-04-25

**Authors:** Catherine A. Risebro, Paul R. Riley

**Affiliations:** UCL Institute of Child Health, 30 Guilford Street, London, WC1N 1EH, UK

**Keywords:** heart development, ventricle, trabeculations, compaction, congenital heart disease

## Abstract

The formation of the ventricles of the heart involves numerous carefully regulated temporal events, including the initial specification and deployment of ventricular progenitors, subsequent growth and maturation of the ventricles through “ballooning” of chamber myocardium, the emergence of trabeculations, the generation of the compact myocardium, and the formation of the interventricular septum. Several genes have been identified through studies on mouse knockout and transgenic models, which have contributed to our understanding of the molecular events governing these developmental processes. Interpretation of these studies highlights the fact that even the smallest perturbation at any stage of ventricular development may lead to cardiac malformations that result in either early embryonic mortality or a manifestation of congenital heart disease.

## INTRODUCTION

The mature adult heart is a complex, vital organ. It consists of four chambers that work synchronously to pump blood and nutrients to the morphologically distinct pulmonary and systemic circulatory systems. The ventricles are responsible for providing the contractile force required to pump the blood away from the heart. Many congenital cardiac abnormalities are associated with defects in ventricular form and function; indeed, the most common forms include ventricular septal defects (VSDs), which reportedly occur at a frequency of between 2 and 6 in 1000 live births[1,2]. Other ventricular-related congenital heart disease (CHD) includes hypoplastic left heart, hypoplastic right heart, and ventricular noncompaction in which the ventricular myocardium is hypertrabeculated[3]. Clearly, it is of great importance to gain a thorough understanding of the mechanisms governing the formation of the ventricles during development in order to better understand the etiology of CHD.

In vertebrates, the heart is the first organ to form in the embryo proper, beginning soon after gastrulation. In the mouse, this occurs at around embryonic day 7 (E7) and around the third week of development in humans. Cardiac precursors reside in the anterior region of the primitive streak either side of the embryonic midline, known as the cardiac crescent (Fig. 1). At around E8.0, the precursors migrate medially from the cardiac crescent to form the primitive linear heart tube along the ventral midline of the embryo (Fig. 1). The linear heart tube consists of an exterior myocardial tube with an endocardial lining separated by an extracellular matrix known as the cardiac jelly, and is attached to the body wall by the dorsal mesocardium. The primitive heart initiates rhythmic contractions and undergoes rightward looping

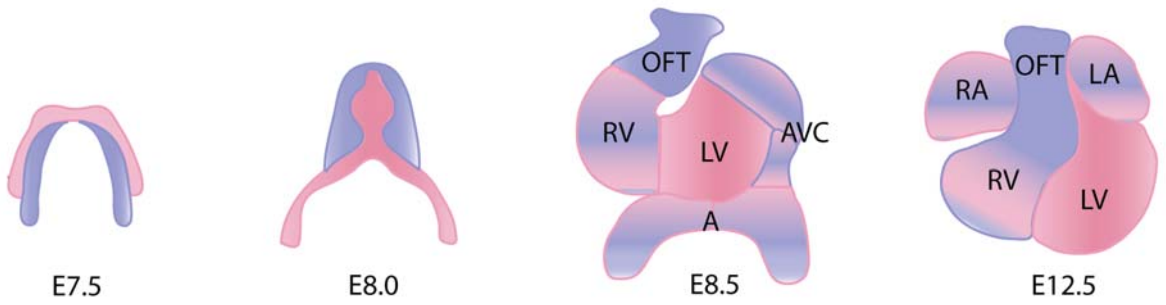

**FIGURE 1.** Morphology of the developing heart and the two sources of myocardial cells. Vertebrate cardiac morphogenesis begins shortly after gastrulation when the primary (classical) heart field progenitors are specified in the anterior, lateral mesoderm forming the cardiac crescent (pink) at E7.5. The second cardiac lineage (blue) is located in the splanchnic mesoderm, medially and dorsally compared to the primary heart field. As the embryo folds, the primary heart field progenitors then migrate and fuse at the ventral midline of the embryo, forming the muscular linear heart tube at E8.0. At this stage, the second lineage precursors become located in a more dorsal and anterior position. Between E8.25 and E10.5, the linear heart tube undergoes rightward looping and the second lineage precursors migrate into the heart, contributing to both the arterial and venous poles of the developing heart. Retrospective clonal analysis suggests that all regions of the heart tube are colonized by both sources of cardiac progenitors with the exceptions of the left ventricle, which is derived entirely from the primary heart field, and the outflow tract, which is formed exclusively from the second lineage. Following cardiac looping, the heart enters the remodeling phase during which the cardiac chambers mature and septate, forming the four-chambered organ shown here at E12.5. LA, left atrium; LV, left ventricle; RA, right atrium; RV, right ventricle; OFT, outflow tract; A, atria. Modified from Kelly et al.[9], ©2001 with permission from Elsevier and from Buckingham et al.[4], ©2005 with permission from Nature Publishing Group.

at around E8.5 in the mouse and day 25 in humans. Coincident with cardiac looping, significant contributions are made to both the arterial and venous poles of the heart from the recently defined second cardiac lineage (Fig. 1). Subsequently, the atrial and ventricular chambers are specified and “balloon out” from the heart tube. The chambers septate and the vessels are orientated to bring the outflow region in between the developing ventricles at the ventral surface, and the inflow region to the dorsal side of the heart. A key morphological transition in ventricular chamber development is the formation of the trabecular and compact myocardium, which enable the developing heart to deal with the increased hemodynamic load essential for pumping blood, nutrients, and oxygen throughout the rapidly growing embryo.

In this review, we will initially provide a brief overview of the contribution of cardiac precursors from the primary and secondary lineages and the initial specification of the ventricular chambers since this has been extensively covered elsewhere[4]. The principal focus will be on the subsequent maturation of the ventricles, including the expansion from the looped heart, and considerable discussion will be devoted to the development of the trabeculated and compact myocardium. Finally, the formation of the interventricular septum will be discussed and extrapolated to the prevalence of ventricular septal defects in CHD. Included throughout are selected examples of the genes and signaling pathways identified as playing a role in each of these developmental processes. We focus primarily on the morphological processes and regulation of murine cardiac development, since the vast majority of informative studies to date have been carried out in the mouse; however, interspecies comparisons are highlighted where either differences have been observed or significant insight has been gained through use of an alternative model system.

## DEPLOYMENT OF CARDIAC PRECURSORS

Initially, cell fate analyses indicated that the linear heart tube was patterned along its anterior to posterior (A/P) axis into segments that correspond to the future aortic sac, outflow tract, right and left ventricles, and atria, respectively[5]. However, with the discovery of the anterior heart field/second lineage, the contribution of the linear heart tube has been shown to be restricted mainly to the left ventricle (Fig.

1)[6,7,8]. Studies in chick and mouse identified a population of myocardial precursor cells in the pharyngeal mesoderm, anterior to the early heart tube, which has been termed the anterior, or secondary, heart-forming field, and is distinct from the primary heart field attributed to the cardiac crescent and linear heart tube[6]. The anterior heart field resides in the splanchnic mesoderm, at the cardiac crescent stage coincident with the precursors of the primary heart field, but is located medially and dorsally to the classical cardiac crescent (Fig. 1)[7,9]. The anterior heart field was shown to give rise to the outflow tract myocardium, a region added after ventricle and inflow tract formation[9] and contributes significantly to the myocardium of the right ventricle in mice during cardiac looping[8].

During looping, the linear heart tube elongates primarily through the contribution of cells from the anterior heart field. Expression and lineage tracing studies on the LIM homeodomain transcription factor Islet1 (Isl1)[7], along with retrospective clonal analysis[10], have revealed that the anterior heart field is, in fact, a subset of a much larger progenitor population that contributes to both the arterial and venous poles of the developing heart (Fig. 1) and also to both left and right ventricles. Intriguingly, the retrospective clonal analysis also pointed to the existence of a common precursor for both myocardial lineages that exists prior to the formation of the cardiac crescent[10]. Although tracking clonal growth of myocardial cells has confirmed the existence of two separate cardiac lineages, the results also suggested that each lineage contributes slightly differently to the developing heart than the originally defined primary and secondary heart fields[10]. These studies indicated that both lineages contribute to all regions of the heart, with the exceptions that the left ventricle was derived entirely from the so-called primary/first lineage and the outflow tract was derived entirely from the now-defined second lineage[10]. These results conflict with genetic fate mapping of *Isl1*-expressing progenitors in the secondary heart field[7] and with a more restricted map using a *Mef2c* (myocyte enhancer factor) anterior heart field enhancer element to drive Cre dependent lacZ expression in the second lineage and its derivatives[11]. Collectively the fate mapping studies suggest that the second cardiac lineage does, in fact, contribute to the left ventricle. However, whereas the *Isl1* progenitors appear to give rise to the majority of the left ventricle, the *Mef2c*-expressing cells within the anterior heart field only give rise to proximal portions of the left ventricle[7,11]. The discrepancy here is attributed to the fact that since *Isl1* is expressed in precardiac mesoderm prior to partitioning of the primary and secondary heart fields, it may mark descendents of both fields, whereas the *Mef2c* enhancer-driven expression is only activated in precardiac mesoderm after the secondary heart field has a distinct identity[11].

Despite the lack of a consensus on the precise origins of cardiac progenitors and the developmental potential of the secondary heart field, it appears that, for the most part, precursors for the right and left ventricles do seem to originate from distinct progenitor pools.

## SPECIFICATION OF THE VENTRICLES

A key stage in the early development of the ventricles is brought about by the process of cardiac looping, which occurs coincidentally with the recruitment of cardiac progenitors from the secondary heart field. As the linear heart tube elongates, it breaks away from dorsal mesocardium and subsequently bends to the right with the onset of cardiac looping. Cardiac looping is conserved across all vertebrate species, reflecting its critical importance in heart development. Looping ensures that the presumptive chambers are in the correct orientation and alignment with respect to the inflow and outflow regions, which will ultimately become the great vessels of the heart. The looping process creates a structure with a single primordial left ventricle that has an inflow and an outflow region that connect to the atria via the atrioventricular canal (AVC) and the outflow tract, respectively (Fig. 1). Abnormalities in cardiac looping, leading to improper alignment of the outflow tract, contribute significantly to human congenital heart defects such as double outlet right ventricle, in which the aorta and pulmonary artery arise from the right ventricle. Malalignment of the outflow tract also results in an obligatory ventricular septal defect (VSD) due to displacement of the ventricular septum at the level of the great vessels[12].

Knockout studies in mice have shown that several cardiac transcription factors are essential for appropriate looping morphogenesis and their loss of function tends also to be characterized by a single, common, hypoplastic ventricular chamber. In mouse embryos lacking the early cardiac homeobox gene *Nkx2.5*, looping is initiated, but the heart tube fails to extend properly and, consequently, the embryos die at around E9–10 from heart failure. *Nkx2.5* mutants form a primordial left ventricle (Fig. 2A,B), however, the ventricular tissue fails to express the basic helix-loop-helix (bHLH) transcription factor *Hand1*, and subsequently does not differentiate into chamber myocardium[13]. This suggests that the primary *Nkx2.5*-null defect is in appropriate differentiation of the left ventricle. In *Isl1* null embryos, the single ventricular chamber that develops (Fig. 2C,D) was identified as the left ventricle by expression of *Hand1*, together with a lack of expression of right ventricular markers[7]. A similar phenotype in terms of an apparent absence of right ventricle is also observed in embryos lacking the forkhead DNA-binding protein, *Foxh1*[14], and *Mef2c*[14,15]. Indeed, *Mef2c* appears to be a target of both Isl1 and Foxh1 (through separate enhancer elements)[14,16]. Interestingly, each of these cardiac transcription factors, *Nkx2.5, Isl1, Foxh1,* and *Mef2c* are also expressed in the anterior heart field and it has been suggested that the lack of right chamber identity in the common ventricle of the mutants arises as a result of a failure of the anterior heart field to be added to the developing heart[11]. In the *Nkx2.5* mutants, there appears to be defective contribution of anterior heart field to the primordial left ventricle (R. Harvey, personal communication), however, at least in the *Mef2c* knockout embryos, anterior heart field cells have been observed in the common ventricular chamber[11], suggesting that looping defects seen in *Mef2c* are not due to a failure of anterior heart field migration into the ventricular chamber, but more likely defective differentiation of these cells once in the ventricle, or a failure in their communication with adjacent tissues.

In the zebrafish, there is only a single functional ventricle and scope for single gene mutants to affect the entire ventricular lineage. The zebrafish *pandora* (*pan*) locus encodes a protein similar to a transcription elongation factor, Spt6[17], which appears to regulate the specification of the ventricular precursors. Homozygous *pan* mutants form only a thin stalk of ventricle-like tissue, rather than a muscular chamber that is attached to a bulbous atrial-like chamber (Fig. 2E–H)[18]. Genetic analysis in zebrafish has revealed that the ventricle seems particularly sensitive to genetic lesions, such that in mosaic screens for mutations that impact on cardiac chamber formation, the vast majority adversely affect the formation of the ventricle[19]. In *acerebellar* mutants, loss of FGF8 signaling results in defective early patterning and induction of cardiac precursors, which is accompanied by a severely dysmorphic ventricle at later stages[20]. Mutations in myocardial transcription factors such as *faust/gata5* and *hands off/hand2*, which in zebrafish appear to play roles in myocardial differentiation and survival, respectively (reviewed in [21]), also result in preferential defects in ventricular formation. In many of these fish mutants, the single atrium is comparatively normal, which has led to the suggestion that atrial fate might be the default state and that additional molecular events are required to form the ventricle. In support of this idea, partial perturbation of ventricular development in the fish following exogenous treatment with retinoic acid (RA) promotes atrial development at the expense of ventricular development[22].

The numerous loss-of-function mouse models and the plethora of zebrafish mutants with heart defects, and more specifically ventricular anomalies, thus illustrate how genes involved in these early stages of cardiac specification are absolutely crucial to the developing heart since mutation can result in loss of one or more chambers, leading to arrest at early stages of embryogenesis.

## EXPANSION OF THE VENTRICULAR CHAMBERS

After looping, the heart tube septates into a four-chambered structure in which the systemic and pulmonary circuits are distinct. Each chamber differs in pattern of gene expression. With notable exceptions (e.g., *Nkx2.5, Isl1, Foxh1,* and *Mef2c*), a number of transcription factors implicated in chamber specification also have chamber-specific expression patterns. For example, the related bHLH factors *Hand1* and *Hand2*, which are expressed predominantly in the left and right ventricles, respectively[23]. In mouse models, loss of *Hand2* results in hypoplasia of the right ventricle[23], while in *Hand1*-null embryos, the left ventricle is

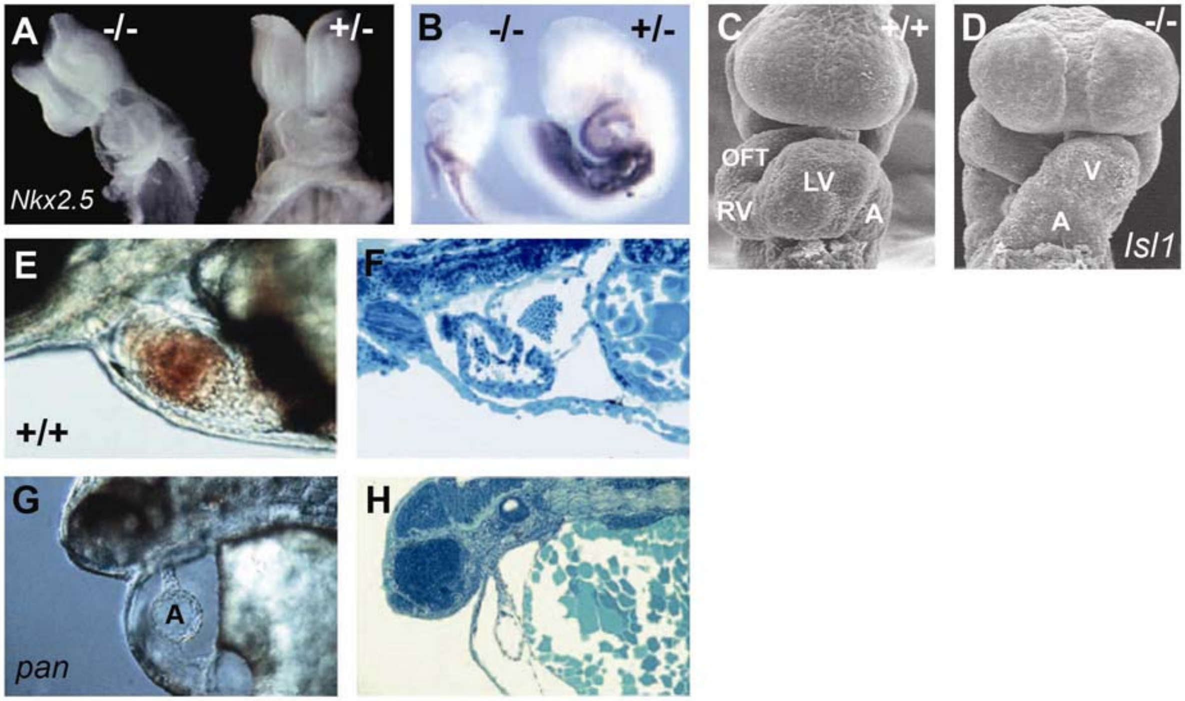

**FIGURE 2.** Specification of the ventricular chambers. The left and right ventricles are, for the most part, formed from distinct cardiomyocyte progenitor pools. The primary heart field/first lineage contributes to the left ventricle, while the second lineage contributes to the right, as demonstrated by the loss-of-function phenotypes for the early cardiac markers *Nkx2.5* and *Isl1*, respectively. (A) Whole mount *Nkx2.5*$^{-/-}$ (left panel) and *Nkx2.5*$^{+/-}$ (right panel) E8.5 embryos. In embryos lacking *Nkx2.5*, the linear heart tube forms, but is morphologically abnormal and does not form ventricular tissue appropriately. (B) *In situ* hybridization analysis for *Hand1* on whole *Nkx2.5*$^{-/-}$ (left panel) and *Nkx2.5*$^{+/-}$ (right panel) embryos. *Hand1* predominantly marks the left ventricle and this expression is lost in the *Nkx2.5*$^{-/-}$ embryo, reflecting a defect in the primary heart field/first lineage. Scanning electron micrographs of wildtype (C) and *Isl1*$^{-/-}$ (D) E9.5 embryos. *Isl1*$^{-/-}$ embryos form only a single ventricular chamber and lack outflow tract and right ventricular components, reflecting a defect in the anterior heart field/second lineage. (E–H) Whole mount (E,F) and sections through (G,H) wildtype (E,G) and *pandora* (*pan*) (F,H) zebrafish mutant embryos (48 hpf). Zebrafish have only a single ventricle, formation of which appears to be regulated by the *pan* locus since *pan* mutants lack ventricular tissue forming only a bulbous atrial-like chamber and outflow tract. LA, left atrium; LV, left ventricle; RA, right atrium; RV, right ventricle; V, ventricle; OFT, outflow tract; A, atrium. A is reproduced from Lyons et al.[13], ©1995; B is reproduced from Biben and Harvey[110], ©1997 with permission from Cold Spring Harbor Laboratory Press; C and D are reproduced from Cai et al.[7], ©2003 with permission from Elsevier; E–H are reproduced from Stainier et al.[18], ©1996 with permission from the Company of Biologists Limited.

hypoplastic[24,25,26]. Ablation of both *Hand* genes results in loss of both ventricles, as observed in both *Nkx2.5*/*Hand2* double homozygous mutants[27] and in a cardiac *Hand1* knockout crossed with the conventional *Hand2* knockout[26]. The double *Hand* knockout phenotype has been likened to the zebrafish *hands off* mutation in which, although more severe in general, no ventricular chamber develops[27,28]. In the chick, however, the *Hand* genes appear to have redundant roles since the loss of a single *Hand* gene does not perturb cardiac development[29]. Redundancy in the chick is facilitated by the fact that, from Hamburger-Hamilton stage 8 onwards, *Hand1* and *Hand2* are uniformly expressed in an overlapping pattern throughout the developing heart, in contrast to only a partial overlap in mice[29]. Moreover, this may reflect a mechanistic difference in the early stages of ventricle formation between mammals and birds. The lack of appropriate ventricle formation in mice mutant for either *Hand1* or *Hand2*, together with the expression of the *Hand* genes at the ventral surface of the linear heart tube and subsequently at the outer curvature of the looped heart, led to the hypothesis that the *Hand* genes may regulate expansion of the ventricular chambers. *Hand* gene dosage appears to be an important consideration here since, in our own studies, we have observed that *Hand1* overexpression, directed exclusively to *Hand1*-expressing cells, results in a small, dense, presumptive left ventricle that has failed to balloon out from the heart tube, which along with a reduction in *Nppa* (encoding atrial natriuretic factor) expression suggests there may be a defect in expansion of the left ventricle and/or differentiation of chamber myocardium (unpublished observations).

Another cardiac transcription factor, the T-box gene *Tbx5*, is expressed in a gradient in the posterior portion of the developing heart, namely the atria, inflow tract, and left ventricle[30]. In addition to severe atrial and inflow tract defects, *Tbx5* knockout mice have left ventricular hypoplasia[31]. Similar defects are also observed in the zebrafish *Tbx5* mutation *heartstrings*[32]. Interestingly, misexpression of *Tbx5* throughout the entire ventricular region in mice inhibits the formation of the right ventricle, as indicated by a lack of *Hand2* expression[33].

The Iroquois homeobox transcription factor *Irx4* is expressed only in the ventricular chambers[34,35], and has been shown to activate *ventricle myosin heavy chain-1* (*VMHC1*) and repress *atrial myosin heavy chain-1* (*AMHC1*) in the presumptive ventricular chambers in the chick[34]. *Irx4* knockout mouse embryos display abnormal ventricular gene expression, including markedly reduced *Hand1* levels, and this precedes cardiomyopathy in the adults, indicating that Irx4 is not essential for ventricular chamber formation, but is important for ventricular function[36].

Previously, the cardiac chambers were thought to arise from specific segments arranged along the A/P axis of the linear and looping heart tube. This view on chamber specification has been significantly revised based on extensive gene expression studies, culminating in the now widely accepted two-step "ballooning model" of chamber formation (Fig. 3A)[37]. The first step is the formation of the linear heart tube, composed of "primary" myocardium, which is polarized in terms of morphology and gene expression along the anteroposterior and dorsoventral axes. The second step is specification of "chamber" (working or secondary) myocardium, which can be first identified at the ventral surface of the linear heart tube and has a distinct transcriptional program. As the heart tube loops, the primordial chambers are aligned facing the outer curvature, from where they subsequently proliferate and "balloon" out[37]. Genes are activated in the outer curvature demarcating the future chamber myocardium of the atria and ventricles including *Nppa* (Fig. 3B,C), *Chisel*, and *Connexin 40* (*Cx40*)[37]. The myocardium of the inner curvature, inflow tract, AVC, and outflow tract retains the molecular signature it had in the linear heart tube as primary myocardium and does not express markers of chamber myocardium.

Growth and expansion of the embryonic ventricles occurs via the addition of myocardial cells (hyperplasia), which is initiated in the early heart tube. Retrospective clonal analysis in the mouse suggests that myocardial growth is orientated and, for the most part, discontinuous between E10.5 through to E14.5 and that clones in each region of the heart expand in distinct and characteristic patterns[38]. In this respect, the two ventricles differ considerably such that clonal growth is circumferential in the embryonic right ventricle, but more complex in the left ventricle showing two perpendicular orientations[38]. This supports the notion of early left/right separation of myocardial precursors and also illustrates differences in the morphogenesis and expansion of the two ventricular chambers such that the right ventricle forms by enlargement of the heart tube, whereas the left ventricle forms by bulging from the outer curvature of the heart tube consistent with the ballooning model of chamber myocardium[37].

Very little is known about precisely how and when chamber myocardium is specified and it is not clear from the studies carried out to date whether chamber myocardium is induced in the linear/primitive heart or whether nonchamber myocardium is actually created from a suppression of the chamber myocardium developmental program. The T-box transcription factor *Tbx2* is expressed in the nonchamber myocardium of the AVC (Fig. 3D,E) and the outflow tract, and prevents chamber formation in these primary regions by repressing chamber myocardium–specific genes such as *Nppa* and *Cx40*[39,40]. Conversely, another T-box transcription factor, *Tbx20*, is expressed throughout the heart in regions of chamber myocardium and represses *Tbx2*, thus permitting chamber formation. Accordingly, *Tbx20* mutants have defects in both the left and the right ventricles[41,42,43,44] in that ventricle-like chambers do form, but chamber myocardium does not differentiate (Fig. 3F,G). This phenotype has been attributed to increased levels of *Tbx2* expression that, in turn, prevents chamber differentiation. The model whereby

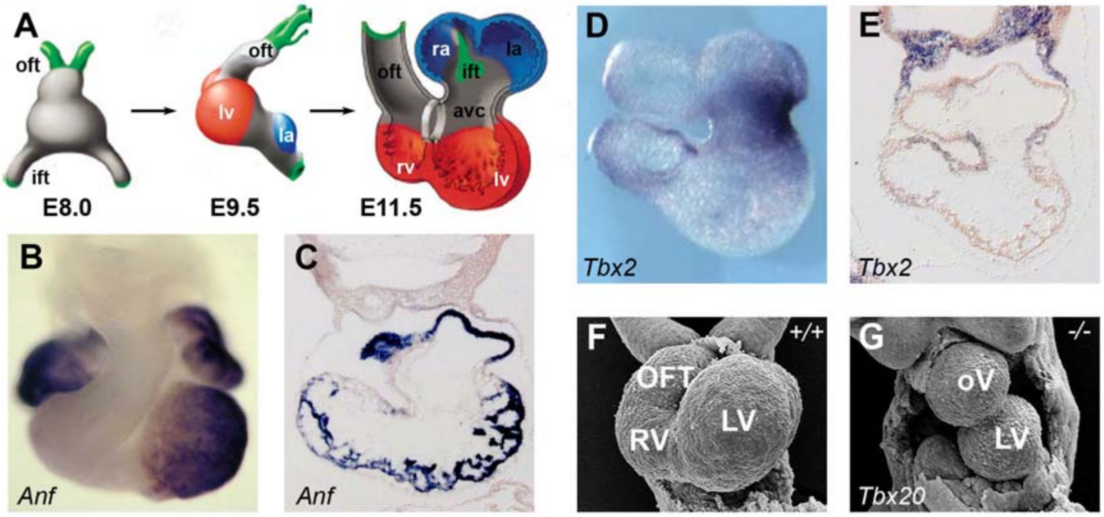

**FIGURE 3.** The "ballooning" model for cardiac chamber expansion. (A) Illustrations of the embryonic mouse heart at E8.0, E9.5, and E11.5 showing primary (nonchamber) myocardium (grey), ventricular chamber myocardium (red), atrial chamber myocardium (blue), and the inflow and outflow tracts (green). Chamber myocardium "balloons" out from the outer curvature of the looped heart tube to generate the atria and ventricles, the inner curvature remains primary, nonchamber myocardium and contributes to the inflow tract, outflow tract, and AVC. (B–E) *In situ* hybridization analysis of whole mount hearts (B,D) and sections (C,E) highlights the mutually exclusive expression patterns of *Nppa* (encoding atrial natriuretic factor; B,C), which marks the cardiac chambers (albeit weakly in the right ventricle), and *Tbx2* (D,E), which marks the nonchamber regions of the heart and represses the formation of chamber myocardium. (F,G) Scanning electron micrographs showing hearts from wildtype (F) and *Tbx20*$^{-/-}$ (G) embryos at E9.0. Tbx20 is required for the differentiation of chamber myocardium. *Tbx20*$^{-/-}$ embryos develop small, abnormal ventricle-like chambers in which chamber myocardium does not differentiate, most likely due to up-regulation of *Tbx2*. LA, left atrium; LV, left ventricle; RA, right atrium; RV, right ventricle; OFT, outflow tract; IFT, inflow tract; AVC, atrioventricular canal; oV, outflow ventricle-like chamber. A is modified from Christoffels et al.[111], ©2004 with permission from Elsevier; B, C, and E are reproduced from Habets et al.[39], ©2002 with permission from Cold Spring Harbor Laboratory Press; D is reproduced from Harrelson et al.[45], ©2004; and F and G from Stennard et al.[41], ©2005 with permission from the Company of Biologists Limited.

Tbx20 represses *Tbx2* to specify chamber vs. nonchamber myocardium appears to hold true for the developing ventricles, but is incomplete with respect to the AVC since Tbx2 has been proposed to repress chamber differentiation in the AVC[45] and yet *Tbx20*, which is essential for chamber differentiation, is also coexpressed with *Tbx2* at high levels in the AVC[44]. This suggests that, despite the fact that Tbx2 is both necessary and sufficient to repress chamber differentiation[40,45], additional factors besides Tbx20 must be required to up-regulate chamber differentiation.

## MATURATION OF THE VENTRICULAR CHAMBERS

### Formation of the Trabeculated Myocardium

Trabeculations begin to form after cardiac looping in both mouse and humans. The ventricular myocardium grows out from the inner, ventral surface in the apical region of the presumptive ventricle, forming trabeculations that are finger-like projections of myocardium, surrounded by endocardium (Fig. 4A). The trabeculations expand rapidly either by myocyte recruitment or by proliferation, and subsequently differentiate. Trabeculations can comprise up to 80% of the cardiac mass[46] and establish the contractile force of the developing mouse heart from E9.5 until around E14.5. The trabeculated myocardium serves as a means to increase myocardial oxygenation prior to the development of the coronary circulation. It also maintains separate blood flow through the embryonic chambers prior to septation[47] and contributes extensively to the development of the conduction system.

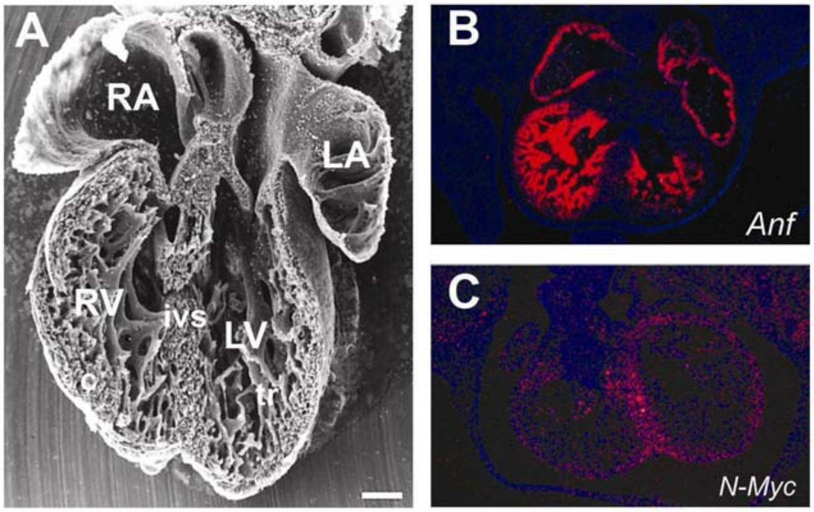

**FIGURE 4.** Maturation of the ventricles. (A) Scanning electron micrograph of an embryonic mouse heart at E14.5 indicating the presence of trabeculated and compact myocardium, and the fully formed muscular interventricular septum (Scale bar = 100 μm). Note that the pattern of trabeculations *ANF* (*Nppa*) does not differ greatly between the two ventricles. (B,C) *In situ* hybridization for *ANF* (B) and *N-myc* (C) on sagittal sections through E10.5 wildtype mouse embryonic hearts. The trabeculated and compact myocardial layers can be demarcated as early as E10.5 by the expression of *ANF* and *N-myc*, respectively. LA, left atrium; LV, left ventricle; RA, right atrium; RV, right ventricle; ivs, interventricular septum; c, compact myocardium; tr, trabeculations. A is reproduced from Sedmera et al.[47], ©2000 with permission from John Wiley & Sons Limited; B and C are courtesy of Edward Morrisey, University of Pennsylvania.

In the mammalian heart, the trabeculations of the left and right ventricles are morphologically distinguishable, in that the trabeculations in the left ventricle tend to be subtly thicker than the right ventricle. In adult humans, but not in mouse, there is a more marked difference in the trabeculations of the respective ventricles, being finer and criss-crossing in the definitive left ventricle, compared to the right[48]. The difference between chick and mammals is more pronounced. In the chick, the trabeculations are thicker and arranged more like a sheet in the left ventricle, but are finer and arranged in a fan-like pattern radiating out from the septum in the right ventricle[47]. In zebrafish, unlike in higher vertebrates, the trabeculations remain at about two cell layers thick, from their early formation (between 72–120 h postfertilization) throughout development, depending entirely on nutrition and oxygenation from the ventricular lumen. Apparent thickening of the trabeculations in the adult fish is thought to be due to cardiomyocyte hypertrophy as the number of cell layers remains the same[49]. The trabeculations are also structurally distinct from those of mammals and birds, adopting a more strut-like appearance with uniform morphology and no apparent regional (apical-basal) differences[49].

Ventricular trabeculation and compaction are closely associated with the regulation of cardiac growth at mid-gestation. Mechanisms controlling ventricular myocardium differentiation, formation of the trabeculations, and their subsequent compaction are poorly understood. Several genes have been identified that appear to play vital roles in these processes (Table 1); however, their precise function and downstream targets and signals remain largely unclear.

Signaling from the endocardium is thought to be required for trabecular growth. In zebrafish *cloche* mutants, the inner endocardial lining does not develop. The myocardial tube remains small and dysmorphic, and trabeculations do not form within the ventricle[50,51]. Mouse embryos lacking neuregulin or its tyrosine kinase receptors ErbB2/B4 have severe defects in ventricular trabeculation (Fig. 5A,B). Neuregulin is secreted by the endocardium and is believed to act at the inner surface of the developing heart, signaling via ErbB receptors expressed in the residing cardiomyocytes to induce their

**TABLE 1**
**Examples of Genes Involved in Key Stages of Ventricular Development**

| Stage Affected | Gene(s) Involved | References |
|---|---|---|
| Initial ventricular specification | Nkx2.5, Isl1, Mef2c, FoxH1 | [7,13,14,15] |
| Ventricular chamber formation | Hand1, Hand2, Tbx2, Tbx5, Tbx20, Nppa | [23,24,31,37,39,41,42,43,44] |
| Trabecular formation | Neuregulin, ErbB2, ErbB4, Serotonin 2B receptor, BMP10, VEGF, Flk1, Ang1, TIE2, SRF, Sema6D, Plexin A1 | [52,53,54,55,56,57,58,59,60, 62,70] |
| Ventricular compaction | RXRα, WT-1, N-myc, FOG2, Epo, EpoR, α4-integrin, TEF1, βARK, Sema6D, Plexin A1, HIF-1α | [67,68,70,72,73,74,75,76,82, 83,85,87,91,92,103,112,113] |

proliferation and trabecular growth[52,53,54]. Similarly, mice that lack the *serotonin 2B receptor* exhibit reduced levels of *ErbB2* and their ventricles lack trabeculations[55]. Other pathways involved in endocardial signaling to the myocardium to induce trabecular proliferation involve vascular endothelial growth factor, VEGF, and its receptor Flk1[56], and Angiopoietin-1 (Ang-1) and its receptor TIE2[57,58,59]. Specific loss of Ang-1 from the myocardium or TIE2 from the endocardium results in marked hypoplasia of the trabeculations, suggesting critical reciprocal signaling between the myocardium and endocardium.

Bone morphogenetic protein (BMP) signaling represents an additional key contributor to ventricular trabeculation; notably, *BMP10* is transiently, but specifically, expressed in the trabeculations between E9.0 and E13.5 after which it becomes restricted to the atria[60]. *BMP10*-deficient mice have hypoplastic ventricular walls and form only primitive trabeculations and consequently die at around E10.5. Lethality at this stage reflects the critical importance of the trabecular myocardium for cardiac function and embryonic development. BMP10 is not required for initiation of the trabeculations, but is essential for their further growth and that of the ventricular wall by maintaining appropriate proliferative activity and preventing premature activation of the negative cell cycle regulator $p57^{kip2}$[60]. Conversely, elevated *BMP10* expression results in hypertrabeculated, noncompacted hearts[60]. Similarly, mouse embryos in which *Nkx2*.5 has been targeted at later stages of cardiac development, using a cre-lox strategy[61], together with conduction defects, have increased ventricular trabeculation and cardiomyopathy with persistent *BMP10* expression in the trabeculations. Furthermore, the morphological defects of these *Nkx2.5* mutant hearts mimic those found in some human patients with *Nkx2*.5 mutations[61].

Maintenance of appropriate myocardial ultrastructure is also essential for cardiac trabeculation and associated compaction. Mice lacking serum response factor (SRF), specifically in cardiomyocytes and vascular smooth muscle cells, reveal poorly developed trabeculations and a thinning compact layer[62]. The failure to form trabeculations in SRF mutant mice (Fig. 5C–F), accounts for their embryonic lethality at E11.5, and is attributed to perturbations in Z-disk formation, sarcomere assembly, and signaling. Additionally, expression profiling and array analyses have revealed the attenuation of key sarcomeric, cytoskeletal, and transcription factors in neonatal cardiomyocytes lacking SRF[63]. These studies suggest a vital role for SRF in the contractile cytoarchitecture necessary for the proper assembly and function of cardiomyocytes within the developing myocardium.

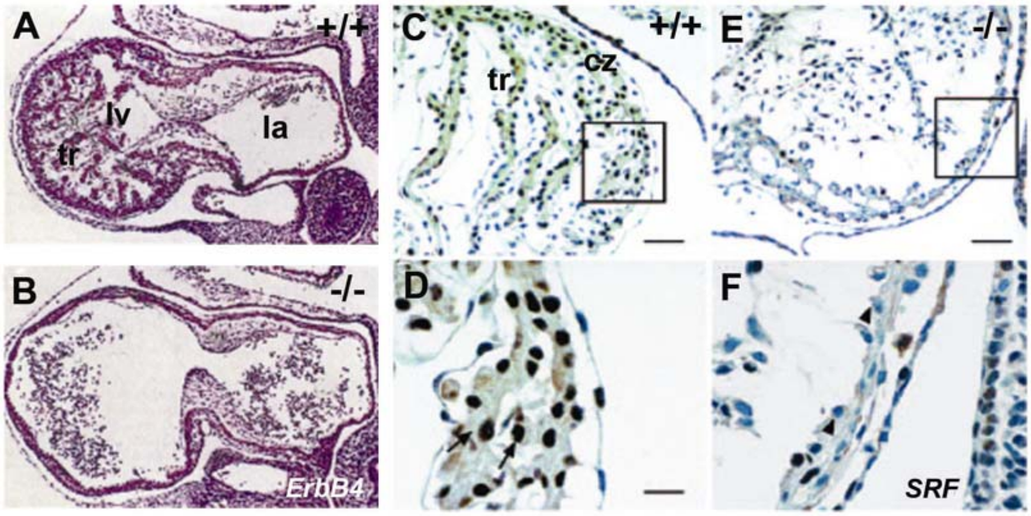

**FIGURE 5.** Development of the trabeculated myocardium. (A,B) Sagittal sections through wildtype, +/+ (A) and *ErbB4*$^{-/-}$ (B) E10.5 embryonic hearts. The neuregulin-ErbB2/B4 signaling pathway is crucial for normal trabeculation of the ventricles; embryos lacking either component fail to develop trabeculations as illustrated by the *ErbB4*$^{-/-}$ mutant (B). (C–F) Immunostaining for SRF on frontal sections through wild type, +/+ (C,D) and mutant, -/- (D,F) hearts at E10.5. D and F represent magnified images of the boxes in C and E, respectively (scale bars = 10 μm), SRF is required to maintain appropriate contractile cytoarchitecture in the developing myocardium, which in turn is essential for the development of the trabeculations. la, left atrium; lv, left ventricle; cz, compact zone; tr, trabeculations. A and B are reproduced from Gassmann et al.[53], ©1995 with permission from Nature Publishing Group; C–F are reproduced from Miano et al.[62], with permission from The National Academy of Sciences USA.

## Formation of Compact Myocardium

During the early stages of ventricular development described above, the compact myocardium is only a few cells thick and much of the required contractile function is provided by the trabeculations. The compact layer is evident (3–4 cells thick) from around E11.5 after which it thickens markedly from E13–14 in the mouse (between 10 and 12 weeks in humans). Growth of the compact layer occurs as trabeculated myocardium begins to compact and become integrated into the ventricular wall (Fig. 4A), concomitant with septation and development of the coronary circulation. Additionally, trabeculated myocardium undergoing compaction contributes to the interventricular septum and also to the papillary muscles and the conduction system[64]. As the compact layer grows, it supplants the trabeculated myocardium in providing the contractile force of the developing heart[65]. From E16 onwards (fourth month in humans), the compact layer comprises most of the ventricular myocardium as it continues to develop and expand, becoming structurally more complex[66]. As a consequence of the increased contractile force required for systemic circulation, the compact layer of the left ventricle is three times thicker than that of the right ventricle in the adult mammalian heart and in birds it is up to five times thicker[47]. Most of the increase in compact myocardial mass in birds is thought to be a result of hypertrophic growth as an adaptation to enhance the blood supply to the flight muscles.

As the outermost portion of the ventricular wall, the compact myocardium is made up of concentrically organized layers of tightly adherent cardiomyocytes. These layers continue to expand throughout gestation and are demarcated by expression of the bHLH/leucine zipper transcription factor *N-myc* (Fig. 4C). N-myc is one of the many genes identified as playing an intricate role in ventricular compaction (Table 1). N-myc is required for cardiomyocyte proliferation and consequently *N-myc* mutant mice die at around E10.5 from heart failure characterized by a failure to form a ventricular septum, thin myocardial wall, and, most notably, a failure of expansion of the compact layer[67,68]. Noncompaction of the myocardium results in serious functional consequences for the heart. In humans, ventricular noncompaction has been found in association with heart failure and sudden cardiac death[69], although it can occur in isolation and is now recognized as a distinct cardiomyopathy[66]. In mice, defects in compact myocardium formation usually result in lethality around E14, reflecting the importance of the compact layer for heart function from this stage of development onwards.

Signaling can occur between myocardial cells in the compact layer and those in the trabeculations to coordinate both compact layer extension and trabeculation. In the chick knockdown of the transmembrane type semaphorin, Sema6D (or its receptor Plexin A1) leads to a thin compact layer and defective trabeculation (Fig. 6A,C,E). Expression of the Plexin A1 extracellular domain can rescue the *Plexin A1* defective trabeculation, but not that resulting from defective *Sema6D* expression (Fig. 6B,D,F; [70]). Since, *Sema6D* and *Plexin A1* are both expressed within the compact layer, whereas myocardial cells in the trabeculations express *Sema6D* alone, this suggests that Sema6D functions both as a ligand and receptor for Plexin A1. Thus “forward” and “reverse” signaling exists between the trabeculated and compact myocardium in the chick to regulate ventricular expansion and trabecular formation. Interestingly, since knockdown of *Sema6D* or *Plexin A1* did not affect either proliferation or apoptosis in the mutant hearts, Sema6D is thought to regulate the motility of myocardial cells, in a manner analogous to its previously reported role in axon guidance[71].

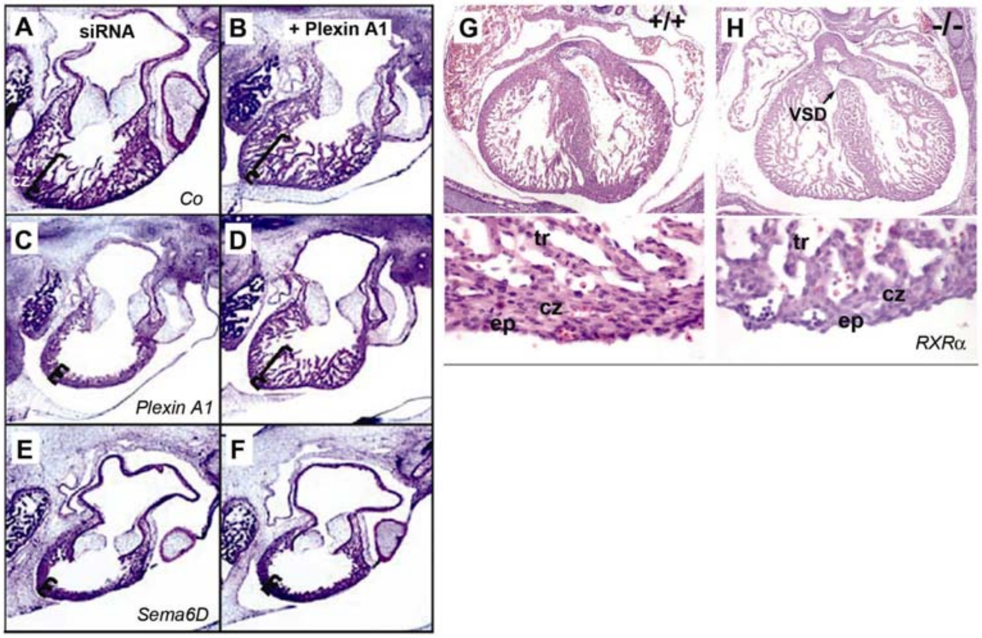

**FIGURE 6.** Development of the compact myocardium. (A–F) Cross-sections through HH-stage 10 chick embryos following electroporation of siRNA constructs *in ovo*. *Plexin-A1*- and *Sema6D*-specific siRNAs suppressed ventricular expansion and both trabeculations and compact myocardium formation (C,E). Injection of Plexin-A1 endocardial cells induces myocardial trabeculation in chick embryos treated with either control (B) or *Plexin-A1* siRNAs (D), but not in embryos treated with *Sema6D* siRNA (F). Sema6D coordinates both compact layer expansion and trabeculation functioning as both a ligand and receptor for Plexin-A1; an example of forward and reverse signaling between compact and trabeculated myocardium to enhance migration of myocardial cells into the trabeculations. (G,H) Frontal sections through wildtype (F) and $RXR\alpha^{-/-}$ (G) E14.5 embryos focusing on the heart. The RA signaling pathway is required for appropriate formation of the compact ventricular myocardium. Consequently, compaction is perturbed in embryos lacking *RXRα* resulting in a hypoplastic ventricular wall, notably with a thin compact layer and an incompletely formed interventricular septum resulting in a VSD (H). co, control; cz, compact zone; tr, trabeculations; VSD, ventricular septal defect; ep, epicardium. A–F are reproduced from Toyofuku et al.[70] with permission from Nature Publishing Group; G and H are reproduced from Chen et al.[84], ©2002 with permission from Elsevier.

Several loss-of-function mouse models, including mice lacking the *retinoid X receptor-α* (*RXRα*)[72,73], do not form a proper compact layer and have a poorly formed ventricular septum (Fig. 6G,H). While factors such as N-Myc act cell autonomously on the myocardium[60], many genes implicated in ventricular compaction appear to work in a non-cell autonomous manner. Wilm’s tumor gene *WT-1*, for example, is not expressed in the myocardium, but in the epicardium, yet mice deficient for *WT-1* exhibit reduced thickness of the compact layer[74].

The epicardium and epicardially derived cells play a crucial role in the development of the ventricular compact layer. Thin myocardium is observed in several mouse models with perturbed epicardial development, including *α4-integrin*[75] and vascular cell adhesion molecule, *VCAM-1*[76]. *α4-integrin* is expressed in the epicardium and *VCAM-1* in the myocardium; a physical interaction of these two molecules is thought to be important for the epicardium to adhere to the myocardium[75]. Myocardial defects have also been observed in chick where epicardial defects inhibited ventricular compaction[77] and, furthermore, placing a physical barrier between the proepicardial organ and heart results in loss of patches of epicardium with the underlying myocardium becoming hypoplastic[78].

It is known that the epicardium plays a critical role in the development of the coronary vasculature concomitant with ventricular compaction. For reviews on coronary vasculature formation, see reference [79] and references within. The coronary vasculature is essential to provide nutrients and oxygen to the thickening myocardial wall. Subepicardial mesenchymal cells (SEMCs) undergo epithelial-to-mesenchymal transformation (EMT) and migrate into the myocardium where they contribute to various components of the coronary vessels including vascular smooth muscle and endothelial cells[80,81]. As mentioned above, mice deficient for WT-1 develop a thin myocardial wall, and they have defects in epicardium formation and subsequent coronary vascularization in that less SEMCs are formed and those that do form fail to migrate into the myocardium[82]. In addition to appropriate epicardial formation, genes involved in EMT and migration of the epicardial-derived cells are crucial for coronary vasculogenesis and myocardial compaction/growth. Friend-of-GATA 2 (FOG-2), a cofactor for the GATA4/5/6 cardiac transcription factors, is essential for formation of the coronary vasculature[83]. In *FOG*-2 mutants, coronary vessels fail to form and the ventricular myocardium is hypoplastic, yet the epicardium develops normally, indicating that the defect seems to be attributable to aberrant EMT[83].

Furthermore, epicardial cells contribute in another way to the maturation of the ventricles by communicating to the underlying myocardium, for example, through RA and the growth factor erythropoietin (Epo)[84,85,86]. Both *Epo* and *Epo receptor* (*EpoR*) knockout embryos exhibit ventricular hypoplasia due to reduced cardiomyocyte proliferation[87]. The mitogen produced by the epicardial cells in response to such signals remains unknown, however, recent data suggest that members of the fibroblast growth factor (FGF) family such as FGF9, and possibly other FGFs, may constitute the RA-regulated (distinct from Epo-regulated), epicardially derived mitogen[88]. Insights from *in vitro* studies have led to the hypothesis that the compaction defect in *RXR*α null mice is caused by a failure of the epicardium to respond to RA in order to produce the trophic factors that induce compact layer proliferation and morphogenesis[84]. Recently, an epicardial-restricted *RXR*α mutant has been generated that suggests epicardial signaling is absolutely required for appropriate cardiac morphogenesis[89]. Furthermore, it has been shown that RXRα signaling is mediated, at least in part, by activation of both PI3 kinase and MAP (Erk) kinase pathways[90]. In this study, Neuregulin signaling (from the endocardium) was also shown to activate the PI3K and Erk pathways to induce cardiomyocyte proliferation, suggesting that epicardial and endocardial signals converge to induce proliferation and yet diverge to induce compact or trabeculated morphologies. Precisely how these signals diverge to reorganize the ventricular chamber into morphologically distinct trabeculated and compact myocardium remains unknown.

Functional feedback is evident in terms of myocardial oxygenation on the formation of the trabeculated and compact myocardium. The developing trabeculations contribute towards vascularization and oxygenation of the expanding myocardium that, in turn, requires a constant supply of oxygen to maintain appropriate contractile function. A mismatch in myocardial demand with oxygen supply can ultimately lead to apoptosis and necrosis, therefore, tight regulation of oxygen-dependent gene expression is required to compensate for any reduction in oxygen availability (hypoxia). Hypoxia-induced transcription factor, HIF-1α, is an important factor required for controlling gene expression in response to oxygen levels[91] activating Epo and VEGF to provide metabolic adaptation under reduced oxygen conditions. Mice lacking *HIF-1α*, specifically in the developing heart, do not have obvious defects in myocardial architecture or ultrastructure, however, the myocardium is compromised functionally in terms of reduced contractility and vascularization[92]. In addition to regulating oxygen homeostasis, the ventricular myocardium is also able to adapt to its changing functional requirements by modifying its proliferative structure. Embryonic cardiomyocytes are capable of self-regulating their proliferative rate in response to mechanical stress, although the cascade of events translating mechanical stimulus into cell division is not fully understood[93]. Trabeculations proliferate more slowly than the outer compact myocardium and show higher degrees of differentiation. This differential growth establishes an appropriate myocardial architecture that, in turn, ensures proper functional loading of the heart. In humans, pathways affecting ventricular growth are likely to underlie the etiology of congenital malformation of the right or left ventricle. Decreased mechanical loading of the left ventricle results in failed left ventricular chamber expansion and ultimately hypoplastic left heart syndrome (HLHS), a rare manifestation of CHD (3.8% of cases), but which accounts for 25% of all CHD mortalities[94]. HLHS is characterized by profound remodeling of the ventricular myocardial architecture and significant reduction in left ventricle myocardial volume. In the chick, a left atrial ligation model of HLHS reveals that myocardial remodeling is accompanied by decreased rates of cell division, elevated apoptosis. and misexpression of FGF/PDGF growth factors[93]. It remains to be determined whether these cellular changes also occur in the pathogenesis of HLHS in humans.

## Interventricular Septum Formation

Following cardiac looping and chamber expansion, there is a requirement for separation of the chambers to ensure unidirectional blood flow and to maintain distinct systemic and pulmonary circulation. In mice, this occurs between E10.5 and E14.5, and in humans, it takes place between weeks 4 and 7. The first evidence of septation of the left and right ventricles is the formation of the primary muscular interventricular septum (IVS; Fig. 4A) that forms from the nonchamber myocardium of the primary heart tube coincident with “ballooning” (for review see [95,96]). Further septation of the chambers is then established by the formation of the endocardial cushions, which are the precursors of the valves and membranous septa[97], at two points along the heart tube, the AVC and the outflow tract.

A degree of uncertainty surrounds the precise cellular origins of the IVS. Retrospective clonal analysis suggests that since clones at the interventricular boundary extend into either left or right ventricular myocardium, the IVS has a dual origin rather than, as previously thought, arising from left ventricular myocardium alone[38]. In contrast, genetic fate mapping suggests that the entire ventricular septum is derived from a population of anterior heart field cells and indeed this may explain the observation in the clonal analysis that myocyte clones are able to span the septal boundary[11].

The IVS is first morphologically apparent from the point of ventricular “ballooning” and has two principal components: an “inlet” portion derived from the structures that septate the atrioventricular junction and which includes the primary muscular septum (trabecular contribution) that develops from the septum between the trabeculated left and right ventricles, and an “outlet” portion derived from the structures that septate the proximal outflow tract[95,96]. As the IVS forms, the apical opening that persists between the two ventricles is termed the interventricular foramen. In humans, the myocardium surrounding this communication between the embryonic ventricles is called the primary ring, which is delineated by expression of GIN (Ganglion Nodosum) epitope[98]. The primary ring remodels as that of the inner curvature of the ventricular segment and expresses appropriate markers of nonchamber myocardium. Remodeling of the primary ring along with appropriate expansion of the AVC permits the separating atria and dividing outflow tract to be shared between the developing apical components of the two ventricles[99]. Closure of the interventricular foramen to prevent interventricular communication is brought about by fusion of the proximal part of the outflow tract cushions with the atrioventricular cushions. This event may either produce an interventricular and atrioventricular part of the membraneous septum, as distinct from the primary muscular IVS[96], or simply an atrioventricular component of the septum depending on the extent of delamination of the septal leaflet of the tricuspid valve[100]. The majority of ventricular septal defects (VSDs) are likely to be muscular arising from a failure in proper development of the primary interventricular septum that, in turn, may be related to noncompaction. However, most muscular VSDs that arise during development tend to close during late gestation or the neonatal period[101]. Human VSDs involving defects in the membraneous portion of the IVS arise from failed closure of the embryonic interventricular foramen and represent the majority of VSDs that require surgical repair[102].

Unlike the mouse where the IVS has a compact arrangement from the outset, the chick IVS appears to form by fusion of trabeculated sheets and is less compact[47]. The human IVS is more compact and thicker than the mouse appearing intermittently to be as thick, if not thicker, than the free wall of the left ventricle[66].

Since the compact myocardium contributes a substantial portion of the IVS, notably to the primary muscular septum, it is perhaps not surprising that many of the genes involved in ventricular compaction also affect muscular IVS development. For instance, in the *RXR*α and the *Epo* knockouts described above the failure to undergo compaction appears to be coupled to VSDs (Fig. 4G). Similarly, mice lacking *endothelin-1* (*ET-1*) have a hypoplastic compact zone and associated high prevalence of VSDs[103].

However, other factors also contribute to the IVS and insight into the roles of these factors has arisen principally from the study of human patients that present with VSDs. In humans, TBX5 has been implicated in IVS formation via studies on Holt-Oram syndrome (HOS) patients. HOS is a rare autosomal disease caused by TBX5 haploinsufficiency and in the majority of patients is characterized by muscular VSDs and/or atrial septal defects (ASDs)[30]. *Tbx5* heterozygote mice provide a good model for the human condition since they exhibit many cardiac anomalies similar to individuals with HOS[31]. The incidence of VSDs implies that Tbx5 may play a role in IVS formation and it has been proposed that the boundary of *Tbx5* expression may contribute to the correct positioning of the IVS, since misexpression of *Tbx5* in the entire ventricular region resulted in a lack of septum formation[33]. Interestingly, it also resulted in ectopic *Hand1* expression throughout the ventricles, which was shown to abrogate septum formation in another study[104]. Moreover, a recent study has shown that patterning of the IVS is regulated by Tbx5 and Sall4[105]. Sall4 is a member of the Spalt-family of zinc-finger transcription factors mutations of which cause Okihiro syndrome (OS) that, in turn, is frequently characterized by VSDs[106,107,108,109]. *Sall4* is positively regulated by Tbx5 and is expressed in the left ventricular myocardium, but most predominantly in the IVS. Tbx5 interacts with Nkx2.5 to induce expression of *Nppa*[31], which as mentioned above is required for formation of chamber myocardium, and, therefore, is repressed in the region of the IVS. Sall4 interaction with Tbx5 in the IVS represses the activation of *Nppa*[105]. Thus, in cases of Sall4 haploinsufficiency, and following *Tbx5* haploinsufficiency where *Sall4* expression is decreased, increased Tbx5-dependent activation of *Nppa* inhibits septum formation.

## CONCLUSIONS

The molecular and morphological data described here provide significant insight into the key events involved in the formation of the ventricles during vertebrate heart development. While much information has already been extrapolated from the use of model organisms, and especially from the study of knockout and transgenic mouse strains, there is clearly still more to learn about the precise regulation of ventricular development. A number of outstanding questions remain to be addressed that fall under the broad categories as outlined in this review. Despite recent advances in the area of patterning of the myocardium, we still require a more precise definition of the different populations of precursor cells that contribute to and specify the ventricles of the heart; notably via lineage tracing and molecular profiling of the primary, secondary, and newly defined common precursor lineages. Understanding how the primary and secondary heart fields merge and whether reciprocal interactions between the two populations control boundary formation between the ventricles also remains to be determined. Precisely how the mechanical processes of looping and subsequent chamber “ballooning” are driven remains an enigma and is a central issue for understanding ventricular morphogenesis. In instances where we have information on individual genes that, when knocked out or overexpressed, result in loss of one or other of the ventricles, impaired trabeculation and/or compaction, or a prevalence of VSDs, there is a further requirement to define the signaling pathways centered around such key factors. Moreover, it is important that we expand current insight into the contribution of signaling between neighboring lineages within the heart and gain a greater understanding of the underlying cellular processes of chamber development, such as the crucial balance between ventricular myocyte proliferation and differentiation, appropriate coronary vasculogenesis and collateral growth.

Finally, an even more significant challenge to the field is to determine specifically how the molecular and cellular mechanisms identified as essential to the formation of the ventricles relate to the disease situation. This is especially pertinent in light of the fact that ventricular anomalies account for the single largest contribution to the etiology of congenital heart disease.

## ACKNOWLEDGMENTS

We thank members of the Molecular Medicine Unit for fruitful discussions and all of the investigators who permitted us to reproduce figure panels as referenced. P.R.R. is supported by the British Heart Foundation and Medical Research Council career establishment award.
